# Sternal Osteomyelitis and Abscess Caused by Elbowing during a Basketball Game

**DOI:** 10.1155/2012/298187

**Published:** 2012-07-03

**Authors:** Hideo Ichimura, Yuichiro Ozawa, Tetsuya Sato, Kanji Matsuzaki, Yuichi Yoshii, Seiji Shiotani

**Affiliations:** ^1^Department of General Thoracic Surgery, Tsukuba Medical Center Hospital, 1-3-1 Amakubo, Tsukuba-shi, Ibaraki 305-8558, Japan; ^2^Department of Cardiovascular Surgery, Tsukuba Medical Center Hospital, 1-3-1 Amakubo, Tsukuba-shi, Ibaraki 305-8558, Japan; ^3^Department of Orthopedics, Tsukuba Medical Center Hospital, 1-3-1 Amakubo, Tsukuba-shi, Ibaraki 305-8558, Japan; ^4^Department of Radiology, Tsukuba Medical Center Hospital, 1-3-1 Amakubo, Tsukuba-shi, Ibaraki 305-8558, Japan

## Abstract

A 15-year-old boy was referred to our hospital for further investigation and treatment of sternal osteomyelitis due to blunt chest trauma, more specifically elbowing during a basketball game 19 days earlier. On an initial presentation, his chest was markedly swollen and chest computed tomography demonstrated a sternal fracture and massive fluid collection in the chest wall. Since his general condition remained fairly good, we initially selected minimal drainage concomitant with antibiotics; if it was unsuccessful, we planned to switch to a more radical debridement procedure. The patient recovered without further invasive intervention and was discharged on postoperative day 26. There is no sign of recurrence six months after operation. This case report indicates that minimal drainage would be a good option for treatment in a phased strategy.

## 1. Introduction

Sternal fracture due to blunt chest trauma rarely develops into infectious complications such as sternal osteomyelitis and mediastinal abscess [1, 2]. We present here a case of sternal osteomyelitis attributable to an unusual cause, namely, elbowing during a basketball game; the case was successfully treated by minimal invasive drainage.

## 2. Case Presentation

**Figure 1:**
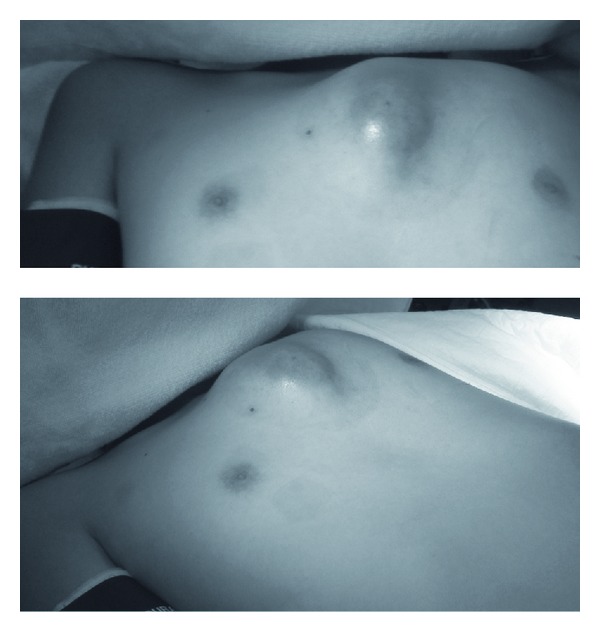
Photographs of the anterior chest region show extensive swelling.

**Figure 2:**
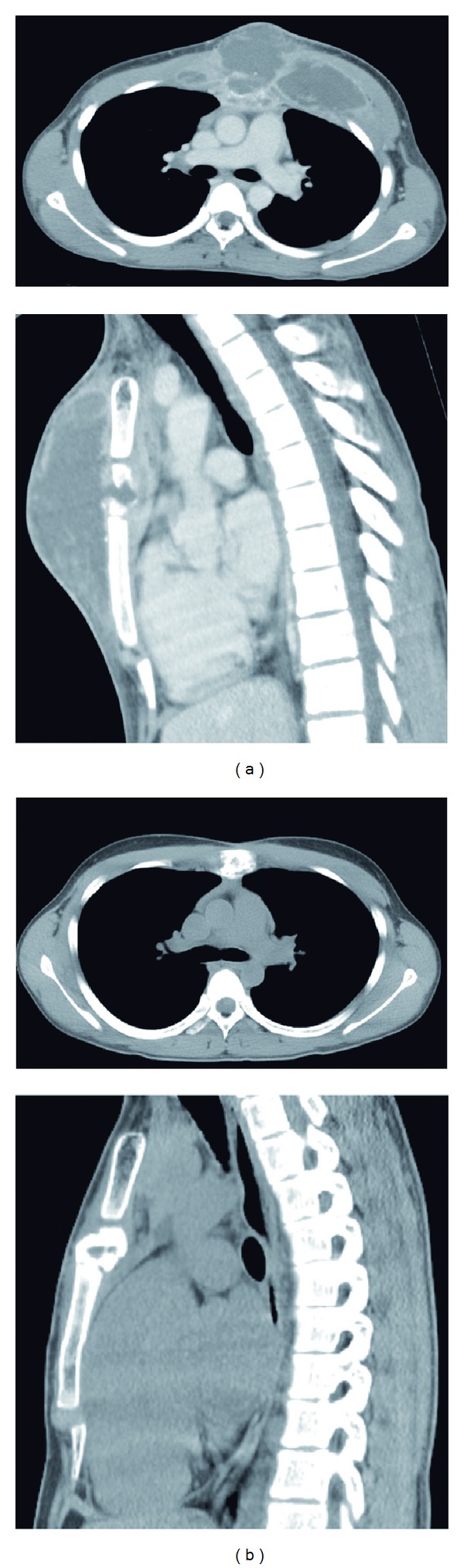
(a) Chest CT scan (upper) and reconstructed sagittal image (lower) revealed a sternal fracture and massive abscess formation. (b) Follow-up CT revealed the disappearance of fluid collection.

A 15-year-old boy without any medical history was referred to our hospital for osteomyelitis of the sternum and a subcutaneous abscess extending over half of the anterior chest wall. The patient's chest had been elbowed by an opponent during a basketball game 19 days earlier. He stated that there were no abrasions or lacerations at the time of injury, but 10 days later, he developed chest wall swelling (Figure 1) and fever. He consulted a local orthopedist and underwent chest computed tomography (CT), leading to provisional diagnosis. The chest mass was punctured, and the pus was drained. Subsequently, the patient was sent to our hospital. Chest CT revealed a sternal fracture and extensive fluid collection in the chest wall (Figure 2(a)). Increased density of the region behind the sternum was also observed, but we concluded that inflammation had not affected the anterior mediastinum. Because the patient did not appear severely ill, we performed pus drainage from a minimal incision and subcutaneously applied a 19 Fr silicone drain connected to a closed drainage system (BLAKE drain and J-VAC Reservoir; Johnson and Johnson Medical, Tokyo) under general anesthesia. The drained material yielded a *Staphylococcus aureus *isolate on microbiological culture; this culture was sensitive to all antibiotics tested. The patient was treated by intravenous administration of sulbacillin for 2 weeks, followed by oral administration of tosufloxacin for 10 days. The drain was removed on postoperative day 14. The patient was discharged on postoperative day 26, and antibiotic therapy was discontinued. While infectious granulation was observed at the drain tube-penetrating site for a few months after discharge, complete epithelialization of the site was confirmed 4 months after the surgery. Follow-up CT performed 4 months after surgery showed disappearance of fluid collection and ossification of the fracture site (Figure 2(b)). The patient did not show any symptoms and signs of recurrence 6 months after the surgery.

## 3. Discussion

As osteomyelitis of the sternum due to blunt chest trauma is rare clinical situation [1, 2], it is necessary to decide a management procedure specific for each condition. The aggressiveness of treatment approach for sternal osteomyelitis varies from medical treatment to radical surgical measure [3–5]. In the present case, his general condition was maintained as “fairly good,” and severe systemic symptoms excluding fever were absent. Therefore, we initially planned minimal drainage; if it was unsuccessful, we planned to switch to a more radical debridement and application of a vacuum-assisted closure system [4, 5]. Fortunately, more aggressive radical intervention was not required for this patient.

When an abnormal infected granulation was apparent at the drain tube site after discharge, we were concerned about his osteomyelitis turning chronic. Chronic osteomyelitis could derive malignant lesions [6]. During the follow-up examination in the outpatient clinic, the granulation was found to have spontaneously regressed, and the wound was closed with complete epithelialization. Although we recognized this condition without any local redness, warmth, and fluctuation as cured, it is necessary to perform long-term followup. This minimally invasive treatment minimized his hospital stay as well as the influence on his school life. Therefore, we believe this would be a good option for treatment in a phased strategy.

## References

[1] Cuschieri J, Kralovich KA, Patton JH, Horst HM, Obeid FN, Karmy-Jones R (1999). Anterior mediastinal abscess after closed sternal fracture. *Journal of Trauma*.

[2] Menash GA, Gold JP, Schreiber T, Isom OW (1988). Acute purulent mediastinitis and sternal osteomyelitis after closed chest cardiopulmonary resuscitation: a case report and review of the literature. *Annals of Thoracic Surgery*.

[3] Omura K, Misaki T, Takahashi H, Kobayashi K, Watanabe Y (1994). Omental transfer for the treatment of sternal infection after cardiac surgery: report of three cases. *Surgery Today*.

[4] Cowan KN, Teague L, Sue SC, Mahoney JL (2005). Vacuum-assisted wound closure of deep sternal infections in high-risk patients after cardiac surgery. *Annals of Thoracic Surgery*.

[5] Eyileten Z, Akar AR, Eryilmaz S (2009). Vacuum-assisted closure and bilateral pectoralis muscle flaps for different stages of mediastinitis after cardiac surgery. *Surgery Today*.

[6] McGrory JE, Pritchard DJ, Ilstrup D, Rowland CM (1999). Malignant lesions arising in chronic osteomyelitis. *Clinical Orthopaedics and Related Research*.

